# Generative Deep Learning Design of Single-Domain Antibodies Against Venezuelan Equine Encephalitis Virus

**DOI:** 10.3390/antib14020041

**Published:** 2025-05-14

**Authors:** Jinny L. Liu, Gabrielle C. Bayacal, Jerome Anthony E. Alvarez, Lisa C. Shriver-Lake, Ellen R. Goldman, Scott N. Dean

**Affiliations:** 1 Center for Bio/Molecular Science and Engineering, US Naval Research Laboratory, Washington, DC 20375, USA; jinny.l.liu.civ@us.navy.mil (J.L.L.); jeromeanthonyealvarez@gmail.com (J.A.E.A.); mrlake@verizon.net (L.C.S.-L.); ellen.r.goldman.civ@us.navy.mil (E.R.G.); 2 Naval Research Enterprise Internship Program, US Naval Research Laboratory, Washington, DC 20375, USA; acbayacal@yahoo.com

**Keywords:** generative AI, nanobodies, viruses

## Abstract

Background/Objectives: Venezuelan equine encephalitis virus (VEEV) represents a significant biothreat with no FDA-approved vaccine currently available, highlighting the need for alternative therapeutic strategies. Single-domain antibodies (sdAbs) present a potential alternative to conventional antibodies, due to their small size and ability to recognize cryptic epitopes. Methods: This research describes the development and preliminary evaluation of VEEV-binding sdAbs generated using a generative artificial intelligence (AI) platform. Using a dataset of known alphavirus-binding sdAbs, the AI model produced sequences with predicted affinity for the E2 glycoprotein of VEEV. These candidate sdAbs were expressed in a bacterial periplasmic system and purified for initial assessment. Results: Enzyme-linked immunosorbent assays (ELISAs) indicated binding activity of the sdAbs to VEEV antigens. In vitro neutralization tests suggested inhibition of VEEV infection in cultured cells for some of the candidates. Conclusions: This study demonstrates how generative AI can expedite antiviral therapeutic development and establishes a framework for quick responses to emerging viral threats when extensive example databases are unavailable. Additional refinement and validation of AI-generated sdAbs could establish effective VEEV therapeutics.

## 1. Introduction

Venezuelan equine encephalitis virus (VEEV) poses a significant risk, due to its potential for causing severe disease in humans and animals, its historical production as a biological weapon in several countries, and its transmission dynamics [1]. After infecting humans through bites by mosquitoes, VEEV can cause disease involving fever, headache, myalgia, and, in severe cases (4–14%), neurological disease [2]. Animal disease can also result in severe illness or death, with higher mortality rates than in humans. It being highly infectious via aerosol inhalation makes it a potential biothreat, in addition to incidences of natural outbreaks, during which thousands of people have become infected.

Work on a VEEV vaccine and therapeutics have been ongoing since the middle of the 20th century. The attenuated TC-83 strain of VEEV, generated by 83 serial passages where it acquired 12 mutations, with one mutation, appearing in amino acid position 120 (T to A) within the envelope E2, affecting the viral adherence and entry into cells. The E2 mutation does not significantly alter its structure, since TC-83 sdAbs binding to viral envelope proteins were able to neutralize both TC-83 and virulent strains in cell cultures [3]. TC-83 is a BSL-2 strain and has been widely utilized for research purposes after its development as a vaccine for laboratory workers and members of the US military [4]. While the TC-83 vaccine is somewhat effective in preventing human disease, a large proportion of recipients do not seroconvert, and evidence in non-human primates suggests that vaccination is not protective against aerosol exposure [4]. Given the lack of an effective FDA-approved vaccine for VEEV, and the absence of any approved vaccine or therapeutic for alphaviruses generally, various therapeutic avenues are being explored, with antibody therapeutics, in particular, being of considerable interest [5].

Monoclonal antibodies (mAbs) have been demonstrated to protect non-human primates against aerosolized VEEV, even 48 h after exposure [6]. Similarly, neutralizing mAbs against the E2 glycoprotein have been demonstrated to protect mice against VEEV aerosol challenge [7]. However, since mAbs are large (~150 kDa) and unstable, as well as difficult and expensive to produce, they are not the best option for treatment, where administration in the field without cold storage may be necessary. Single-domain antibodies (sdAbs, also known as nanobodies) are a promising alternative. Derived from the unique heavy chain-only variable domains of camelids, these small (~15 kDa) antibodies bind with similar affinity and specificity to mAbs, while having greater access to hidden epitopes due to their smaller size and superior thermostability [8]. Although sdAbs have short serum half-lives due to their small size, genetic linking to an anti-albumin sdAb is a proven method to extend serum half-life while maintaining a relatively smaller reagent [9]. Importantly, sdAbs have previously been shown to bind to and neutralize various viruses, including the alphaviruses Chikungunya and VEEV [3,10]. In caplacizumab, a sdAb treatment for acquired thrombotic thrombocytopenic purpura, which was approved by the FDA in 2019 [11], there is evidence that sdAb therapeutics can achieve approval.

Given their growing popularity, with numerous examples accumulating in databases of sequences, and, critically, their size and relative ease of production, sdAbs have recently become a target for the demonstration of protein design using generative deep learning models. These generative models have the ability to greatly speed up the discovery and design process that is normally used in the identification of sdAb candidates. Two groups have shown that the inverse folding utility of the Evolutionary Scale Model (ESM-IF1) could be used to design sdAbs with high affinity to specific targets [12,13]. Several groups have used generative pre-trained transformers (GPTs) or trained their own transformers based on GPT-2 or similar models to generate novel, high-quality antibody sequences [14,15,16]. Many of these very recent reports have shown promising results in (1) the generation of novel sdAb sequences, (2) the demonstration of functions (e.g., affinity to a targeted epitope) reliant on very accurate structure predictions, and (3) the improvement of existing antibodies (whether affinity, stability, or other features). However, none of the previous work has been applied toward VEEV, usually focusing on applications with larger available datasets, such as SARS-CoV-2. In line with these approaches, we investigated whether these methods could be developed toward the problem of VEEV binding and neutralization.

In this report, we present the design and characterization of VEEV-binding sdAbs produced by a generative AI model. Utilizing a dataset of known alphavirus-binding sdAbs, the model generated sequences with high predicted affinity and specificity for the E2 glycoprotein of VEEV. These AI-designed sdAbs were subsequently produced in *Escherichia coli* and purified for characterization. ELISA indicated binding activity of the sdAbs, and neutralization assays suggested inhibition of VEEV infection in cell cultures. This research explores the potential application of generative AI in antiviral therapeutic development and may contribute to approaches for addressing emerging viral threats, potentially offering an avenue for future antiviral drug discovery and development against VEEV and other alphaviruses.

## 2. Materials and Methods

### 2.1. Data and Sequence-Generating Model

The model we fine-tuned was the 738 million-parameter ProtGPT2 model produced by Ferruz et al. [17]. This model and its associated functions were obtained from Hugging Face libraries: transformers, datasets, and evaluate [18,19]. To fine-tune the model, we produced a dataset composed of 62 sequences previously shown to bind either VEEV or Chikungunya virus (CHIKV). The VEEV or CHIKV binding sdAbs were found in publications and patents [3,20] (see Supplementary Materials for sequences). This set was augmented by a further 183 sequences previously identified as binding SARS-CoV-2 from the Oxford CoV-AbDab: the Coronavirus Antibody Database [21], totaling 245 sequences. The addition of these nonrelevant sdAb sequences was required for fine-tuning, as the total of 62 sequences was found to be insufficient for this step. This set of 245 was modified by removing non-canonical amino acids (e.g., X or B) and His-tags. The dataset was then randomly split in half (123:122), into training and validation sets. Fine-tuning was performed using the Hugging Face causal language modeling script [19]. Fine-tuning was evaluated over a range of different learning rates (1 × 10^−2^, 5 × 10^−2^, 1 × 10^−3^, 5 × 10^−3^, 1 × 10^−4^, 5 × 10^−4^, 1 × 10^−5^, 5 × 10^−5^, 1 × 10^−6^, and 5 × 10^−6^) and epoch (3 and 4) values. We determined that the model fine-tuned with a learning rate of 5 × 10^−4^ and three epochs had the highest accuracy, lowest loss, and lowest perplexity (Figure S1).

Moving forward with the 5 × 10^−4^ learning rate and three epoch model, we performed inference optimization for the generation of sequences. Here, random combinations of top k, top p, and repetition penalty were used for generation totaling, to an output of 44,062 novel sequences and 441 different parameter combinations. Wasserstein distance between amino acid frequency distributions of the sequences generated from a given top k, top p, and repetition penalty combination, and the combined training–validation set, was used to sort the output to identify the optimal generation conditions (Figure S2). Using this information, top k = 350, top p = 1.0, and repetition penalty = 1.9 was selected as a good set of conditions for generation, with amino acid frequency distributions largely matching between sets (Figure S2).

### 2.2. Binding Prediction and Final Selection

Predicted structures for the top 250 sdAb sequences generated by the fine-tuned model were produced in combination with VEEV E2 using AlphaFold2-multimer via ColabFold with default parameters [22] (see Box 2 in this reference for parameters). Only the top-ranked models for each sequence were moved into binding constant prediction and other analyses. For predicting dissociation constants from predicted structure complexes, Prodigy (v2.1.4) [23] was used with default parameters. Ramachandran plots were generated by overlaying the identified phi and psi angles from the predicted structures with the standard distributions identified by Lovell et al. [24]. Features such as length and isoelectric point (pI) were calculated using the Peptides package in R [25]. NCBI BLASTp version 2.13.0+ was used to evaluate sequence similarity, run against the training and test sets of sequences or nr (all non-redundant GenBank CDS translations + PDB + SwissProt + PIR + PRF, excluding environmental samples from WGS projects), with the max target sequence set to 100, the expect threshold set to 0.05, and scored using BLOSUM62, with compositional matrix adjustment. From the top 250 sdAbs with predicted binding values calculated by Prodigy, the top ten were found using Ramachandran plots, and two were found to have significant outliers, which produced the final list of eight sdAbs for production. The expressed protein sequences are listed and aligned in Figure S3. Clustal Omega (v1.2.4) [26] or MultAlin (v5.4.1) [27] was used for alignments. UpSet plots were produced using the R package UpSetR (v1.4.0) [28].

### 2.3. Protein Production

For protein production, generated sequences (Table S1) were appended with a C-terminal tag (DDDDK) as a means of lowering the pI and assisting in solubility (Table S2; Figure S3). Genes were ordered from Genscript in the pET22b periplasmic expression plasmid with a C-terminal His-tag. The sdAbs were grown and purified as previously described [3,20]. Briefly, sdAb expression plasmids were transformed into *E. coli* Tuner (DE3) for protein production. Overnight cultures were started by inoculating a single colony into 50 mL of Terrific Broth (TB) with 100 µg/mL ampicillin, grown at 25 °C. The next day, the 50 mL cultures were poured into 450 mL of TB with 100 µg/mL ampicillin, grown for 2 h at 25 °C, and then induced by the addition of IPTG to a final concentration of 0.5 mM and grown for a further 2 h, as previously described [3,20]. SdAbs were purified from the periplasm using an osmotic shock protocol, followed by immobilized metal affinity chromatography and fast protein liquid chromatography, as described previously [3,20]. Briefly, after centrifugation, cells from each large culture flask were homogenized in 14 mL of Tris-Sucrose buffer (100 mM Tris, 0.75 M sucrose pH 7.5), and then exposed to 28 mL of 1 mM EDTA, added dropwise while shaking. After 15 min, 1 mL of 0.5 M MgCl_2_ was added and the mixture was incubated for another 15 min, prior to pelleting the spheroplasts. The supernatant was incubated with 0.5 mL of Ni Sepharose, washed, and then eluted with buffer containing 250 mM imidazole. Protein was then further purified by size-exclusion chromatography using a Bio-Rad Enrich SEC70 10 300 column. Traces are shown in Figure S4. SdAb; concentration was determined by UV absorption and stored at 4 °C or at −80 °C for long term storage.

### 2.4. Enzyme-Linked Immunosorbent Assays

The ELISAs assessed the binding of biotinylated sdAbs to irradiated virus and no-virus controls (wells to which no irradiated virus had been added). The sdAbs were biotinylated in PBS using a 10-to-1 molar ratio of EZ-Link sulfo-NHS-LC-LC (sulfo-N-hydroxysulfosuccinimide-long chain-long chain) biotin (Thermo) to sdAb. After incubating the sdAb and sulfo-NHS-LC-LC biotin for about an hour at room temperature, the biotinylated sdAbs were separated from free biotin using a Zeba spin column (Thermo Scientific, Waltham, MA, USA). The concentration of biotinylated sdAb was calculated using the absorbance at 280 nM.

Irradiated VEEV (strain TC-83) was coated onto the wells of a 96-well plate (Maxisorb; Thermo Scientific) at a concentration of 20 µg/mL (diluted into PBS) and allowed to absorb to the surface at 4 °C overnight. The concentration of the irradiated virus preparation used in the ELISA had been previously determined by a Pierce BCA protein Assay Kit [3]. At least sixteen wells (2 columns) were coated for each sdAb tested. The next day, the coating solution was discarded, and wells were blocked for 1 h with PBS supplemented with 4% milk powder at room temperature. In addition to the coated wells, an equal number of blank wells was also blocked as no-antigen controls. After blocking, wells were washed three times with PBST (PBS plus 0.05% Tween 20). A 2-fold dilution series of biotinylated sdAb at concentrations from 20 to 0.3125 µg/mL was prepared in LowCross buffer (Candor Bioscience, Wangen, Germany). The sdAb dilutions, along with a buffer-only control, were added to the plate such that each concentration was added in duplicate (or triplicate) to coated and uncoated wells. The plate was incubated at room temperature for 1 h and then washed, as before. After washing wells with PBST, horseradish peroxidase (HRP)-conjugated Streptavidin was added into wells at a concentration of 1 µg/mL at room temperature for 1 h. After a final wash, peroxidase substrate, SureBlue TMB-1 component (SeraCare, Milford, MA, USA) was added to each well, and the absorbance at 650 nM was read on a Tecan Spark plate reader.

### 2.5. Plaque Reduction Neutralization Tests

SdAbs were evaluated for their ability to reduce plaque formation and neutralize the TC-83 vaccine strain of Venezuelan equine encephalitis virus (VEEV) in Vero cells (ATCC, Manassas, VA, USA). The TC-83 strain used for these experiments can be handled under biosafety level 2 conditions. To measure the range of sdAb concentration for 50% plaque reduction, multiple neutralization assays were performed. For the first neutralization experiment, eight two-fold serial dilutions of each sdAb were prepared, starting at a concentration of 80 µg/mL. Based on the first percentage of plaque reduction result, the sdAb concentration was further adjusted to the range to reach 50% plaque reduction. Then, 125 µL of each sdAb dilution was incubated with 125 µL of virus suspension containing approximately 200 plaque-forming units (PFUs) for 1 h at room temperature. Following the incubation, for cell infection, 100 µL of each sdAb–virus mixture (in duplicate) was added to Vero cells seeded in 6-well culture plates. Plates were incubated at 37 °C for 1.5 h, with gentle shaking every 15 min to allow for virus absorption. After incubation, a 0.6% agarose overlay in 1X MEM (Temin’s modification) (Thermo Fisher Scientific, Carlsbad, CA, USA) was added to each well. Plates were incubated at 37 °C in a humidified 5% CO_2_ atmosphere for 18–20 h. A second 0.6% agarose overlay, containing neutral red at 80 µM in 1X MEM, was then added. After 20–24 h of staining with neutral red, plaques were counted. The percentage of plaque reduction for each well, adding sdAb dilution, was calculated by comparing to plaque numbers with PBS only. The 50% and 80% plaque reduction neutralization sdAb concentrations (PRNT_50_ and PRNT_80_) were obtained from experimental values or calculated by plotting sdAb concentration vs. percent reduction in Excel (v16.96) to estimate the value. Percentages of plaque reduction were obtained from duplicate samples. PRNT_50_ and PRNT_80_ values were calculated from duplicate samples in two independent experiments to determine the standard deviation (SD).

## 3. Results and Discussion

**Figure 1:**
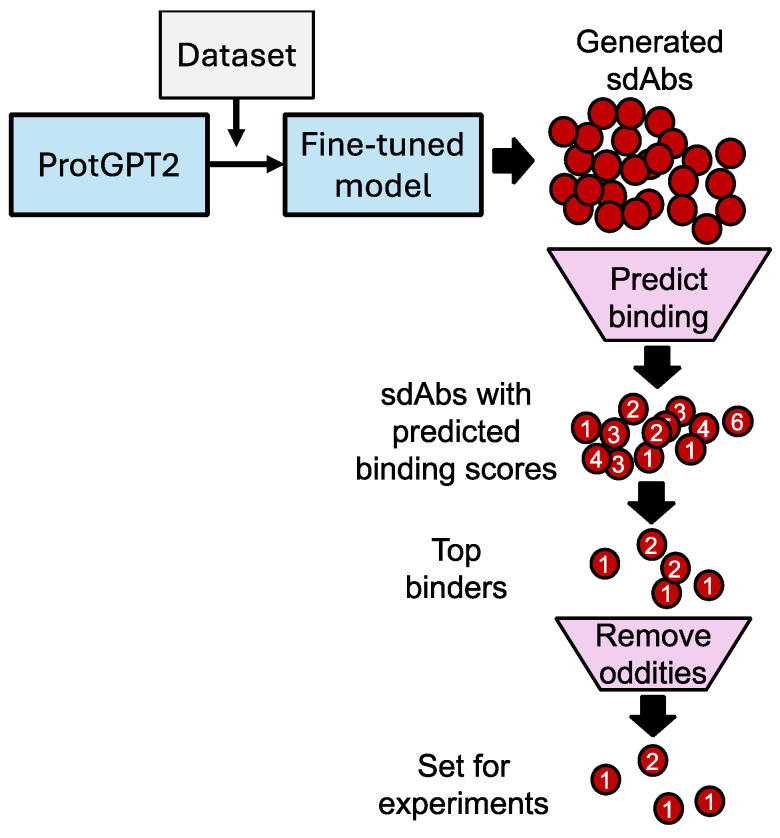
Schematic. Fine-tuned ProtGPT2 with a dataset composed of 62 sequences previously shown to bind either VEEV or Chikungunya virus; this set was augmented by 183 sequences previously identified as binding SARS-CoV-2. Predicted structures for the top 250 sdAb sequences generated by the fine-tuned model were produced in combination with VEEV E2 using AlphaFold2-multimer via ColabFold [22] with default parameters (represented by red circles). Only the top-ranked models for each sequence were moved into binding constant prediction and other analyses. For predicting dissociation constants from predicted structure complexes, Prodigy [23] was used with default parameters (represented by numbers on red circles). Likely unrealistic models, or otherwise sdAbs that may problematic during production, were removed. Ramachandran plots were generated by overlaying the identified phi–psi angles from the predicted structures, with standard distributions identified by Lovell et al. [24]; features such as length and pI were calculated using the Peptides package in R [25]. NCBI BLASTp was used to evaluate sequence similarity. From the top, 41 sdAbs with predicted binding values as calculated by Prodigy; Ramachandran plots were used to down select to the final list of eight sdAbs for production.

Generative AI can accelerate the discovery and design of sdAbs with high specificity and affinity for selected antigens. The standard approach, utilizing animal immunization, can take on the order of weeks or months to obtain new antibodies against a target antigen. Our described process to design novel sdAbs takes approximately 24 h (using an Nvidia RTX 3070 GPU (Nvidia, Santa Clara, CA, USA) to arrive at a selected set of candidate sdAb sequences to produce. However, it is important to note that the more time-consuming, traditional methods of sdAb discovery are required for the function of these new generative AI-based techniques, as a supply of sequences that can be utilized not only for fine-tuning generative models, but also for the major dependence that state-of-the-art protein structure prediction models have on multisequence alignments of natural sequences and experimentally determined structures. Our ProtGPT2 model was fine-tuned using 245 sdAb sequences, including 62 known alphavirus-binding sdAbs (details provided in the Methods section). Augmentation of the fine-tuning set was found to be necessary, as the set of 62 alphavirus-binding examples was found to be insufficient to generate sdAb-like sequences. Using the fine-tuned model, 599 unique sequences were generated. This set of unique potential sdAbs was shown to have an amino acid frequency distribution that was close to that of the training set (Figure S2) once parameter optimization was performed. These sequences then underwent further filtering. (Our schematic for this process is shown in Figure 1).

For antibodies known to neutralize alphaviruses, the E1 and E2 glycoproteins are the most prominent targets, where the specific epitopes involved in the binding of several mAbs (both neutralizing and non-neutralizing) are documented. These include 22 mAbs against E2 alone for various alphaviruses listed in a recent review [5]. Although many of the 62 alphavirus-binding sdAbs used in the fine-tuning set have undetermined epitopes (or unknown protein targets), much of the evidence points to E2 in CHIKV and VEEV for the sequences included in our dataset [29]. Thus, when evaluated for binding in silico, E2 from VEEV was used. In addition, to keep the process less computationally expensive and time-consuming, E2 was utilized, as, although the glycoproteins E1/E2/E3 form a complex, the total number of amino acids summed to >1000 amino acids, a set which was too large for our available hardware.

After selecting the best 250 sequences generated, which were labeled “a1”–“a250”, using pI and other general physicochemical features to down select, the selected sdAbs were paired with E2 in AlphaFold2-multimer predictions. This output was subsequently input into Prodigy for binding affinity prediction. The predicted binding output from Prodigy was used to sort the generated sdAbs (shown in Figure S5). We had previously identified that the PRNT_50_ values from Liu et al. (*n* = 8) were positively correlated with Prodigy-predicted binding affinity to E2, with Rank 1 models produced by AlphaFold2 assessed at R^2^ of 0.71 (squared Pearson correlation coefficient; Figure S6). Using these Prodigy values, considering only the top-ranked models (those with the highest predicted local distance difference test score), we selected those sdAbs that bound to E2 with predicted dissociation constants of <1 × 10^−11^ M. This resulted in a reduction from 250 to 41 candidate sdAbs.

**Figure 2:**
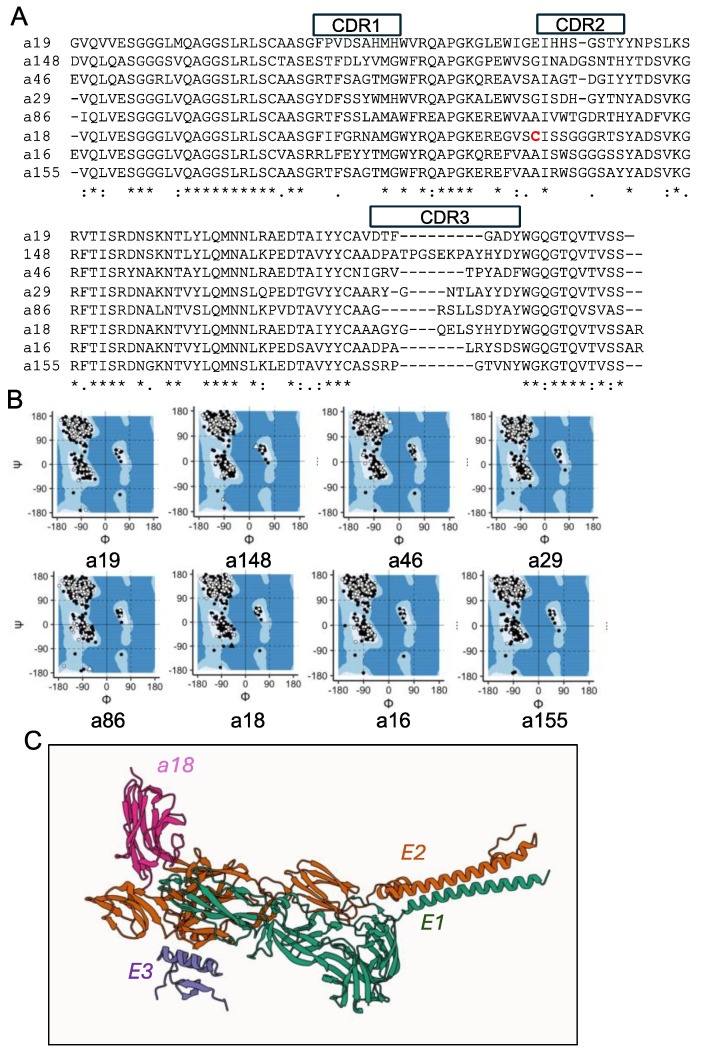
Generated sequences and structures of select sdAbs. (**A**) Sequence alignment. Alignment was performed using Clustal Omega [26]. “*” indicates amino acids with perfect homology; “:” indicates high homology; “.” indicates low homology. CDR regions are defined using the IMGT definitions. Unpaired cysteine is highlighted in red. Protein sequences shown are what was produced. (**B**) Ramachandran plots for each of the sdAbs, modeled in complex with VEEV E2 glycoprotein. Colors of points are indicative of whether the phi–psi angle is part of the sdAb or E2 (white for sdAb and black for E2). Shape indicates whether the phi–psi angle falls within the normal range or is an outlier (circle for normal and triangle for outliers). (**C**) Protein–protein interaction. Predicted structure of representative sdAb a18 (pink), with E1 (green), E2 (orange), and E3 (purple) as ribbon models.

For the final in silico evaluation, we looked at the predicted structures of these final 41 sdAbs using BLAST and Ramachandran plots. While we confirmed that each of the generated sequences was unique (≥1 residue difference) relative to the training and test sets, we double checked that our generated sequences were very different from known sdAbs in the literature and databases. At this stage, no sdAbs were found to be duplicates or previously identified in the literature. BLASTp showed that percent identity of the generated sdAb sequences with those present in GenBank ranged between 73 to 83, and the number of mismatches between these top alignments was >21 in each case, with the a46 being the most unique (Table 1, Figure 2A; BLASTp results are provided in Supplementary Materials). For each sdAb, the framework regions are present in the database, with either positives or exact matches. As expected, CDRs are more variable and unique relative to those in the Oxford SAbDab database [30]. For example, a155 has a top hit in SAbDab of 67% sequence identity; when assessing CDRs (PDB ID 6WAQ), a18 has a top hit of 58% (PDB ID 8ZNZ), and a19 has a top hit of 53% sequence identity (PDB ID 9J3L). We found a similar result for the CDR3s specifically, which could be due, in part, to the relatively short lengths of the CDR3s, e.g., seven amino acids for a19 and eight amino acids for a155 (see Supplementary Materials). Overall, these results suggest that these generated sdAb sequences were not available prior to this study.

Some of the outputs from AlphaFold2-multimer, like structures deposited in the Protein Data Bank, can include Ramachandran outliers, or backbone torsion angles beyond the normal bounds, which can cause issues with either solubility during production or in downstream functional evaluation [24]. By analyzing Ramachandran plots (Figure S7), we determined that some of the predicted structures contained too many outliers for further inclusion, reducing the set of sdAbs moving forward from 41 to 10. Only sdAbs-E2 complexes with ≤1 Ramachandran plot outliers were still included. Certain sdAbs that initially looked promising based on Prodigy-predicted dissociation constants were removed at this stage, including a38, whose rank 1 AlphaFold2-multimer structure had a predicted 1.3 × 10^−15^ M, as it was found to have two distant outliers outside of the acceptable regions. After removing two more sdAbs that had single outliers furthest from the standard regions, we had our final eight, in which practically all backbone torsional angles of all residues were well constructed, supporting higher confidence in the AlphaFold2-multimer models. Ramachandran plots for these eight are shown in (Figure 2B and Figure S7).

Following our selection of the final eight sdAbs, we designed pET22b expression vectors for protein production. The protein yields obtained were between 0.4 mg/L (a19) and 11 mg/L (a148) for sdAbs a16, a18, a19, a86, a155, and a148. The expressed protein sequences, including two previously selected binders, V2B3 and V3G9, were aligned in Figure S3. Despite containing a non-conserved and unpaired cysteine (highlighted in Figure 2A), a18 was produced in sufficient quantities for experiments. In previous work, we found that generative AI-designed sdAbs were insoluble a significant proportion of the time, which led us to add DDDDK (D4K) to the C-terminus in order to push down the pI to a normal range and increase solubility to increase protein yields [31], which we again applied here. By comparing pI with and without D4K among these predicted sdAbs, we found that D4K decreases all pI values toward more acidic values, including previously selected VEEV binders, V2B3 and V3G9, which expressed well without D4K (Table S2). D4K worked well in most of the predicted sdAbs, except a29 and a46, which were still insoluble or produced at insufficient quantities, likely due to aggregation.

**Figure 3:**
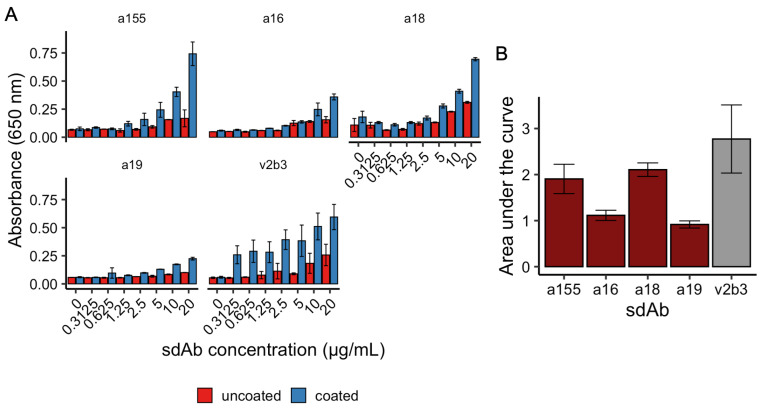
ELISA. ELISAs of four sdAbs (V2B3 as positive control) with readings taken at 650 nm. (**A**) a155, a16, a18, a19, and V2B3 were evaluated for binding to VEEV TC-83 at a range of concentrations from 20 to 0.3125 µg/mL in uncoated or coated wells. (**B**) Area under the curve was calculated for each dose response. a155, a16, a18, and a19 (red) plotted against V2B3 (gray). Bars indicate the means of ≥3 replicates and error bars indicate standard deviations.

Going forward with the remaining six sdAbs, we ran ELISAs to assess binding to irradiated VEEV TC-83. In initial testing, both a86 and a148 were found to bind poorly to killed TC-83 and were excluded from further investigation, leaving four candidates: a155, a16, a18, and a19. In a subsequent ELISA, using known binder V2B3 from Liu et al., 2022 as a control [3], a18 and a155 were found to be the most consistently promising binders to killed virus, with wells coated with killed VEEV showing significant binding at concentrations above 0.625–1.25 µg/mL of sdAb relative to uncoated controls (*p* < 0.05, Student’s *t*-test; Figure 3A). Using the area under the curve to assess all concentrations, although both a18 and a155 were found, at their means, to bind less strongly than the llama-derived V2B3, both had overlapping error ranges with the control sdAb and were found to not be statistically different (*p* > 0.05, Student’s *t*-test; Figure 3B). In contrast to a18 and a155, both a16 and a19 were found to have low or middling binding to killed virus in the ELISA experiments. Finally, we identified in these ELISAs that the top two binding sdAbs, a155 and a18, were also the two sdAbs (out of the eight synthesized) with the highest numbers of predicted contacts output by AlphaFold2-multimer and Prodigy at 142 and 135, respectively (Table 1). While there are not enough data to calculate correlation between the ELISA result and predicted number of contacts with E2, this may be an additional metric to make note of in future investigations for designing sdAbs against VEEV.

With both a18 and a155 showing promising binding, the four clones were assessed by PRNT to determine their abilities to neutralize VEEV strain TC-83. The results shown in Table 2 indicate that each showed some ability to neutralize VEEV compared to positive control, V2B3, and negative control, V3G9. All of the PRNT_50_ values represented 50% reductions in plaque numbers compared to plaque numbers without sdAb (with PBS only). Unsurprisingly, in context with the ELISA results, a18 and a155 showed the best neutralization, while a16 and a19 were the weakest. Compared to VEEV-binding sdAbs previously identified by Liu et al. 2022 [3], and compared to those discovered from llama immunization and phage display library methods, the four sdAbs assessed here are relatively weak neutralizers. As AlphaFold2-multimer is known to have only 30% accuracy for antibody–antigen structure prediction correctness, some low-quality predictions were expected [32,33]. This was compounded by the lack of data for VEEV in particular, where only *n* = 8 sdAbs were available to assess the usability of the Prodigy predictions. In addition, since we modeled the binding via sdAb paired with only E2 glycoprotein, we cannot expect the more complex real-world environment in intact viruses, which includes E1, E3, and other factors, to be well-represented in the model. However, sdAbs that required 2–10 times more to neutralize VEEV TC-83 were also identified by Liu and colleagues from traditional methods, suggesting the model presented here is not without value.

Finally, to assess the possible models of a18 and a155 binding to E2 and VEEV, we interrogated the AlphaFold2-multimer-predicted interface between sdAb and the target (Table 1 and example of a18 with E1/E2/E3 shown in Figure 2C). Here, we saw that, for our small set of sdAbs, the simplest explanatory value for binding was likely to be the predicted number of contacts, where a155 and a18 had 142 and 135 predicted contacts, respectively, with the majority of contact residues being from the CDR3 loop of the sdAbs. Each of the other six sdAbs in this study was predicted to have fewer contacts, suggesting that, in this study, we could have sorted sdAbs by their predicted number of contacts and arrived at our top two generated sdAb sequences, though this relationship is not likely to be retained if more than eight sdAbs were produced.

In order to assess how these sdAbs are modeled in the context of a larger, more realistic complex on the virus surface, we produced models against E1/E2 as well as against E1/E2/E3 (Figure S8). Here, when a18 and a155 were compared to the V2B3 positive control (known to both bind and neutralize VEEV TC-83), we see relatively minor differences in predicted epitope. However, a larger difference is apparent when compared to the negative control, V3G9. Here, when modeled with E1/E2, V3G9 binds where E3 normally sits in the complex—specifically, at a hydrophobic patch—and only makes contacts with E2. In contrast, V2B3, a18, and a155 each bind at a similar location and interact with both E1 and E2. As V3G9 is known to not bind, this could be viewed as a known mechanism for false positives in AlphaFold2-multimer and AF3, where weak binders are modeled at surface-exposed hydrophobic sites and result in incorrect epitope predictions [32]. When relating the interaction sites of these sdAbs in AlphaFold predictions in complex with E1/E2/E3 to those residues identified by Kafai et al. as critical for neutralizing in anti-VEEV mAbs, we see that, while V2B3, a18, and a155 each have one residue included in the critical position list (either K222 or Q225), V3G9 has no overlap with the critical position list [7] (Figure S9 and Supplementary Materials for models). It is, however, unclear whether these predictions are correct without extensive experiments, including cryo-EM or crystallography. If the predictions are taken at face value, binding at K222 or Q225 may act to inhibit either attachment or egress [7].

While this study was in its final phases, AlphaFold3 and open source AlphaFold3 clones like Boltz-1 were released [34]. AlphaFold3 in particular suggests an increased accuracy of ~60% for antibody–antigen interactions, compared to the 30% for AlphaFold2-multimer, suggesting that the success of this and similar studies could be improved by utilizing new and more accurate models. Similarly, in the time since this study was in progress, more promising generative AI models for sequence generation have been released, including ESM3 [35]. In addition, several impressive models for predicting and/or improving the solubility of proteins, such as MPNN_sol_, have been released [36]. The latter may be of particular importance, as two out of the eight designed sdAbs in this study (a29 and a46) were found unproduceable due to their lack of solubility or high aggregation. In future work, we will apply or incorporate these newer models and methods into the nanobody design pipeline.

## 4. Conclusions

As there has been documented emergence of resistance to mAbs and antivirals quickly after the release of the product with many other viruses, including HIV-1, HCV, and, most recently, SARS-CoV-2 [37], the rapid design of novel neutralizing antibody therapeutics is essential to maintain a functioning arsenal against pathogens. While immunization-based methods will likely continue to produce superior products relative to generative AI-based techniques in the short term, due to the need for their rapid design for existing and emerging pathogens and other targets, given sufficient data, we believe protein language models and other competing generative models hold a good deal of promise and may be valuable tools for sdAb development in the future.

## Figures and Tables

**Table 1:** sdAb characteristics.

Name	Length	Molecular Weight (Da)	Isoelectric Point	Ramachandran Normal Count ^1^	Ramachandran Outlier Count ^1^	Number of Contacts with E2 ^2^	Dissociation Constant ((M) at 25.0 °C) ^2^	Highest Percent Identity ^3^
a155	118	12,591	9.9	536	1	142	5.40 × 10^−12^	83
a18	125	13,400	8.8	543	1	135	2.90 × 10^−13^	80
a19	117	12,674	6.9	536	0	119	9.70 × 10^−13^	78
a46	117	12,632	9.7	535	1	119	9.40 × 10^−12^	73
a29	120	13,175	6.5	538	1	118	1.40 × 10^−12^	77
a86	120	12,959	8.7	538	1	115	2.80 × 10^−12^	77
a16	120	13,106	9.0	539	0	115	5.10 × 10^−12^	80
a148	125	13,442	5.0	543	1	106	3.90 × 10^−12^	75

^1^ Whole complex; ^2^ Prodigy prediction; ^3^ BLASTp output (see Supplementary Materials).

**Table 2:** TC-83 PRNT values with sdAbs.

sdAb	TC-83 PRNT_50_ (µg/mL)	Standard Deviation (µg/mL)
a18	39	1.41
a16	53.25	9.55
a155	44	8.49
a19	62.5	3.54
V2B3 *^1^(+)	0.95	0.42
V3G9 *^2^(−)	351	96.17

*^1^ positive control. *^2^ negative control.

## Data Availability

The original contributions presented in this study are included in the article and Supplementary Materials. Further inquiries can be directed to the corresponding author.

## Supplementary Materials

The following supporting information can be downloaded at: https://www.mdpi.com/article/10.3390/antib14020041/s1, Table S1: Sequences of synthesized genes; Table S2: pI values with/without DDDDK added; Figure S1: Fine-tuning optimization: loss, perplexity, and accuracy; Figure S2: Inference optimization; Figure S3: Alignment of expressed sdAb sequences; Figure S4: Traces from size-exclusion chromatography; Figure S5: AlphaFold-multimer–Prodigy results; Figure S6: PRNT_50_ and predicted dissociation constant of sdAbs against VEEV E2 using AF2m models; Figure S7: Ramachandran plots; Figure S8: Model comparison between sdAbs; Figure S9: Overlap of residues of E2 critical for neutralizing anti-VEEV mAbs, and those in the AlphaFold models.

## Abbreviations

The following abbreviations are used in this manuscript:

VEEV	Venezuelan equine encephalitis virus
ELISA	Enzyme-linked immunosorbent assays
SdAbs	Single-domain antibodies
CHIKV	Chikungunya virus
