# Supplementary material for: Generative Deep Learning Design of Single-Domain Antibodies Against Venezuelan Equine Encephalitis Virus
Source: Antibodies (Basel). 2025 May 14;14(2):41. doi: 10.3390/antib14020041 (PMC12101358; doi:10.3390/antib14020041)
Supplement: Supplementary file 1 [file antibodies-14-00041-s001.zip › antibodies-3602770-supplementary.pdf]

Supplementary.

Table S1. AI-generated sdAb sequences.

Name	Sequence
a18	MAVQLVESGGGLVQAGGSLRLSCAASGFIFGRNAMGWYRQAPGKEREGVSCISSGG GRTSYADSVKGRFTISRDNAKNTVYLQMNNLRAEDTAIYYCAAAGYGQELSYHYDY WGQGTQVTVSSAR
a19	MQGVQVVESGGGLMQAGGSLRLSCAASGFPVDSAHMHWVRQAPGKGLEWIGEIH HSGSTYYNPSLKSRVTISRDNSKNTLYLQMNNLRAEDTAIYYCAVDTFGADYWGQG TQVTVSS
a29	MAVQLVESGGGLVQAGGSLRLSCAASGYDFSSYWMHWVRQAPGKALEWVSGISDH GYTNYADSVKGRFTISRDNAKNTVYLQMNSLQPEDTGVYYCAARYGNTLAYYDYW GQGTQVTVSS
a86	MAIQLVESGGGLVQAGGSLRLSCAASGRTFSSLAMAWFREAPGKEREWVAAIVWTG DRTHYADFVKGRFTISRDNALNTVSLQMNNLKPVDTAVYYCAAGRSLLSDYAYWGQ GTQVSVAS
a148	DVQLQASGGGSVQAGGSLRLSCTASESTFDLYVMGWFRQAPGKGPEWVSGINADGS NTHYTDSVKGRFTISRDNSKNTLYLQMNALKPEDTAVYYCAADPATPGSEKPAYHYD YWGQGTQVTVSS
a16	EVQLVESGGGLVQAGGSLRLSCVASRRLFEYYTMGWYRQAPGKQREFVAAISWSGG GSSYADSVKGRFTISRDNAKNTVYLQMNNLKPEDSAVYYCAADPALRYSDSWGQGT QVTVSSAR
a155	MAVQLVESGGGLVQAGGSLRLSCAASGRTFSAGTMGWFRQAPGKEREFVAAIRWSG GSAYYADSVKGRFTISRDNGKNTVYLQMNSLKLEDTAVYYCASSRPGTVNYWGKGT QVTVSS
a46	EVQLQASGGRLVQAGGSLRLSCAASGRTFSAGTMGWFRQAPGKQREAVSAIAGTDG IYYTDSVKGRFTISRYNAKNTAYLQMNNLRAEDTAIYYCNIGRVTPYADFWGQGTQV TVSS

Table S2. pI values with/without DDDDK added.

sdAb	*pI*(with DDDDK)*	*pI*
**a18**	6.64	8.48
**a19**	6	6.53
**a29**	5.82	6.42
**a86**	6.35	7.88
**a148**	5.47	6.02
**a16**	6.66	8.64
**a155**	8.6	9.47
**a46**	8.08	9.3
**V2B3**	6.31	8.08
**V3G9**	5.93	6.72

* pI calculation based on the expressed protein sequences with 6xHis tag.

DISTRIBUTION STATEMENT A. Approved for public release: distribution is unlimited.

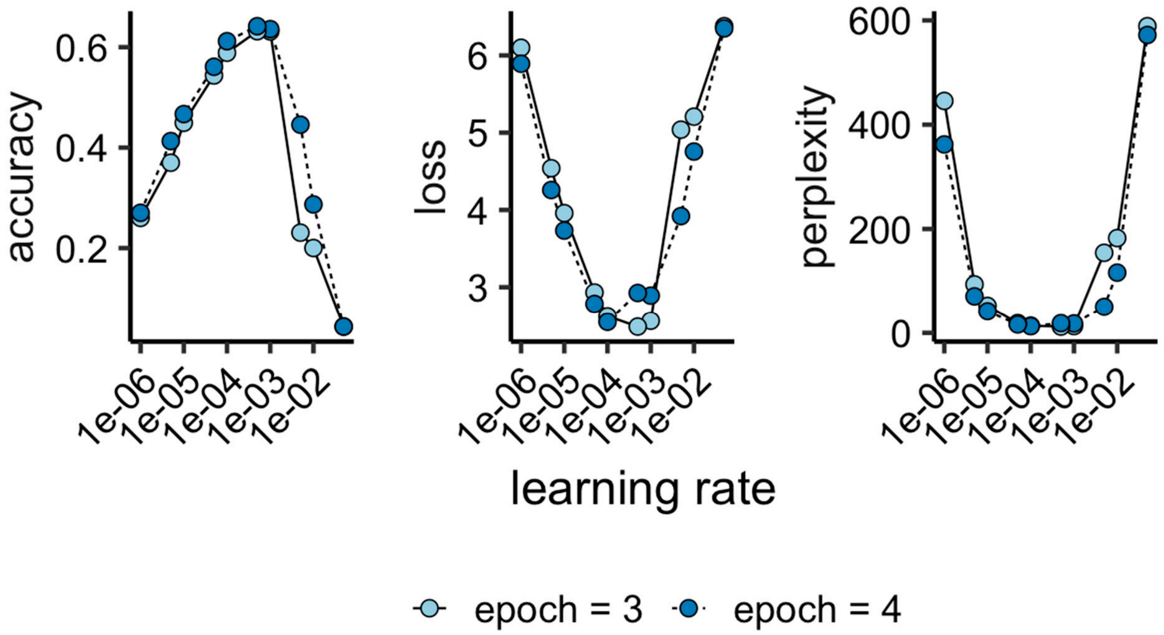

Figure S1. Fine-tuning optimization. Loss, perplexity, and accuracy. Fine-tuning was evaluated over a range of different learning rates and epoch values. We determined that the model fine-tuned with a learning rate of 5 x 10^{-4} and three epochs had the highest accuracy, lowest loss, and lowest perplexity.

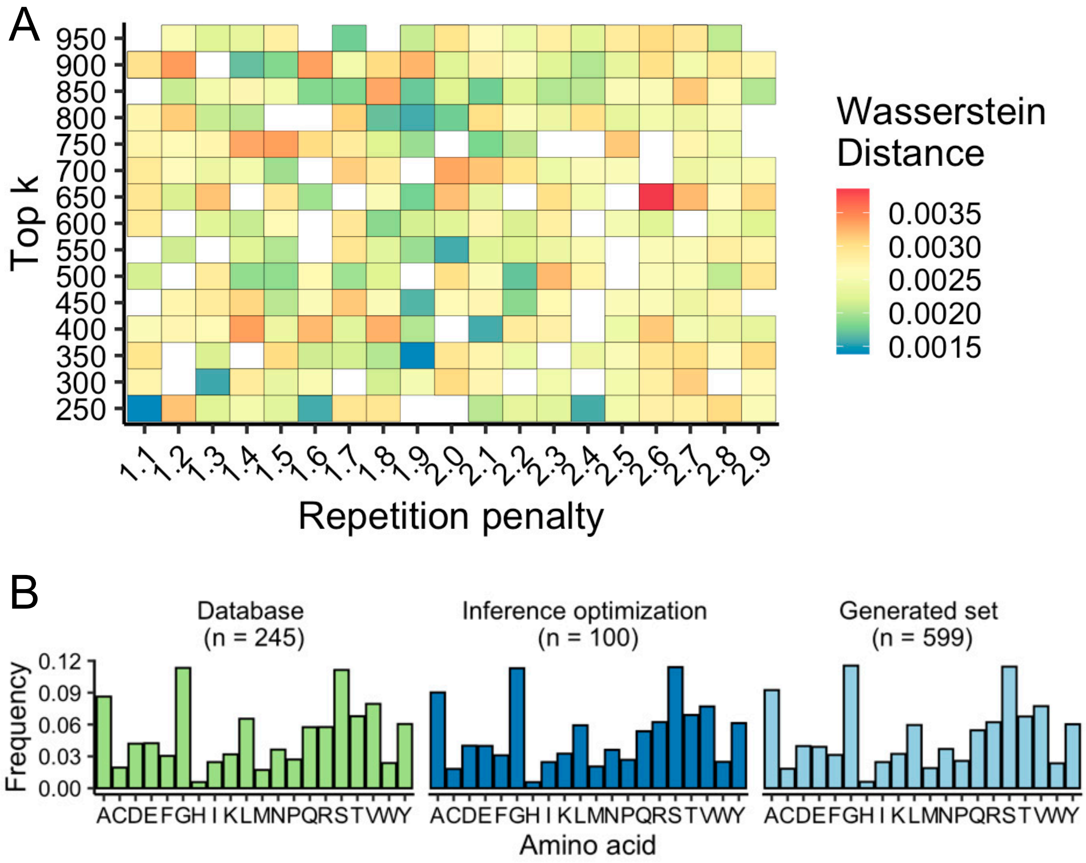

Figure S2. Inference optimization. A) Wasserstein distance between amino acid frequency distributions of the sequences generated from a given combination of randomly selected top k and repetition penalty was compared to the training-validation. Low Wasserstein distance (indicated by blue tile color) indicates more similar amino acid frequency distributions. The additional parameter utilized for optimization, top p, is not shown. B) Amino acid frequency distributions of the database used for fine-tuning (n=245, green), inference optimization (n=100, dark blue), and generated set (n=599, light blue) are shown. The inference optimization and generated sets used the following parameters: top k = 350, top p = 1.0, repetition penalty = 1.9, which was selected as the best set of conditions for generation with amino acid frequency distributions largely matching between sets, based on Wasserstein distance in panel A.

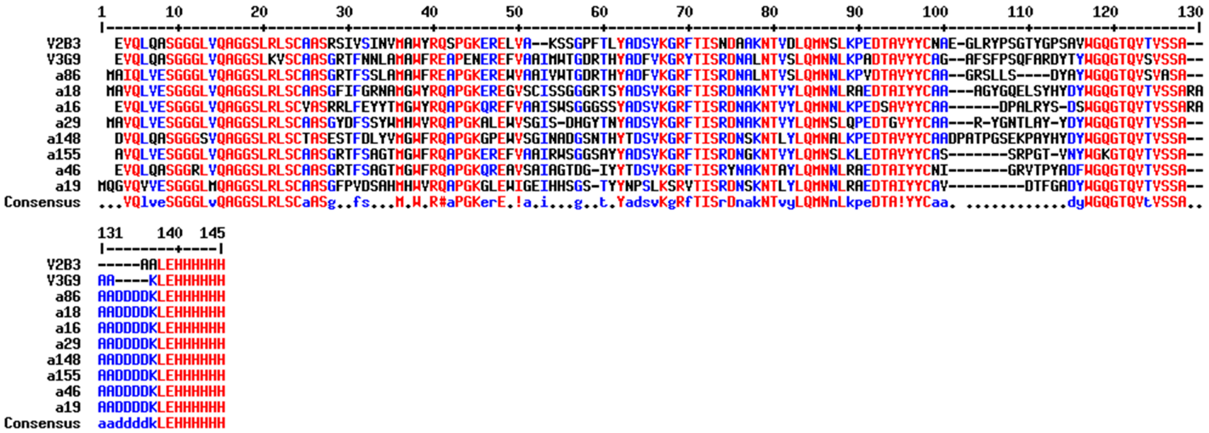

Figure S3. Alignment of expressed sdAb sequences. 6X-His tag was added to C-terminus for protein purification. DDDDK was added for improving solubility characteristics (see Methods for details). The alignment was produced with MultAlin [27].

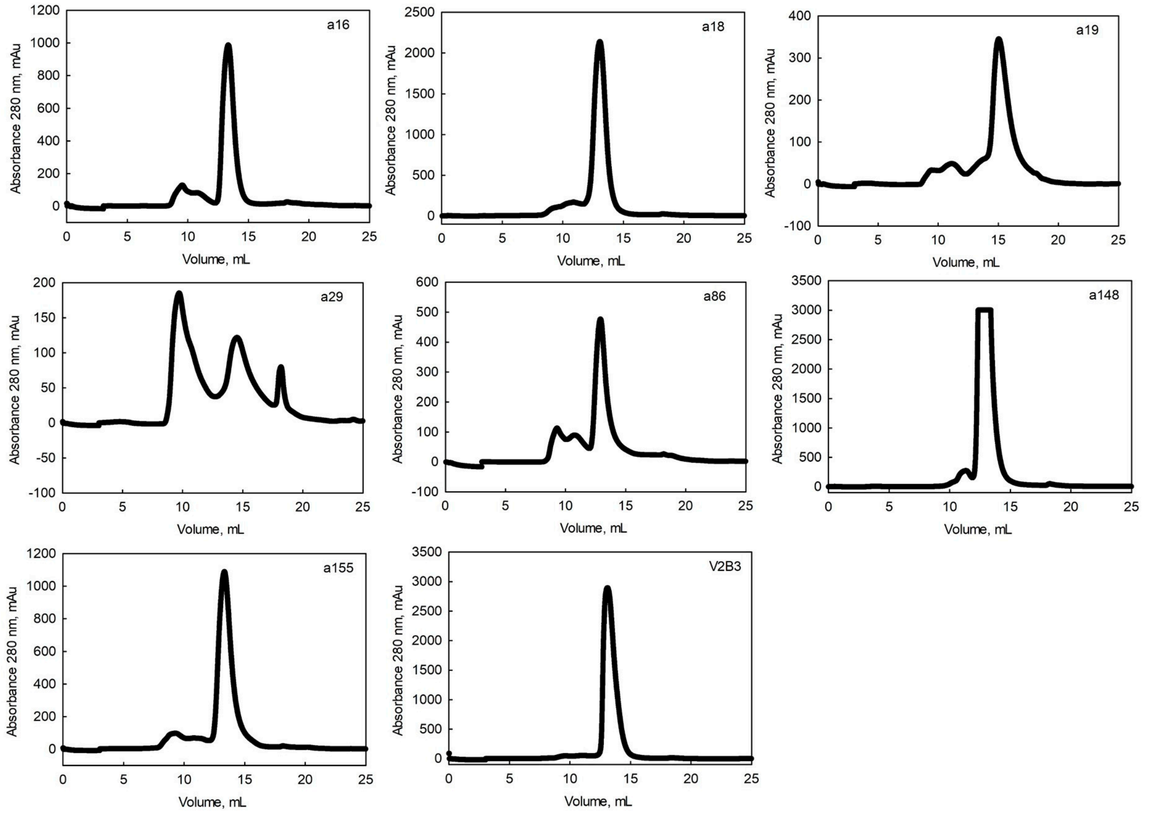

Figure S4. Traces from size exclusion chromatography. After purification by immobilized metal affinity chromatography (IMAC), each sdAb was further purified into PBS by size exclusion chromatography using a Bio-Rad Enrich SEC70 10 300 column. Traces (absorbance at 280 nm) are shown for purifications of seven of the sdAb generated through this study as well as the previously described clone V2B3. Clone a29 has no major peak where we expect for sdAbs; however, the rest show clear signal from the produced sdAb. Each protein was produced at least twice and showed similar purification profiles and yields.

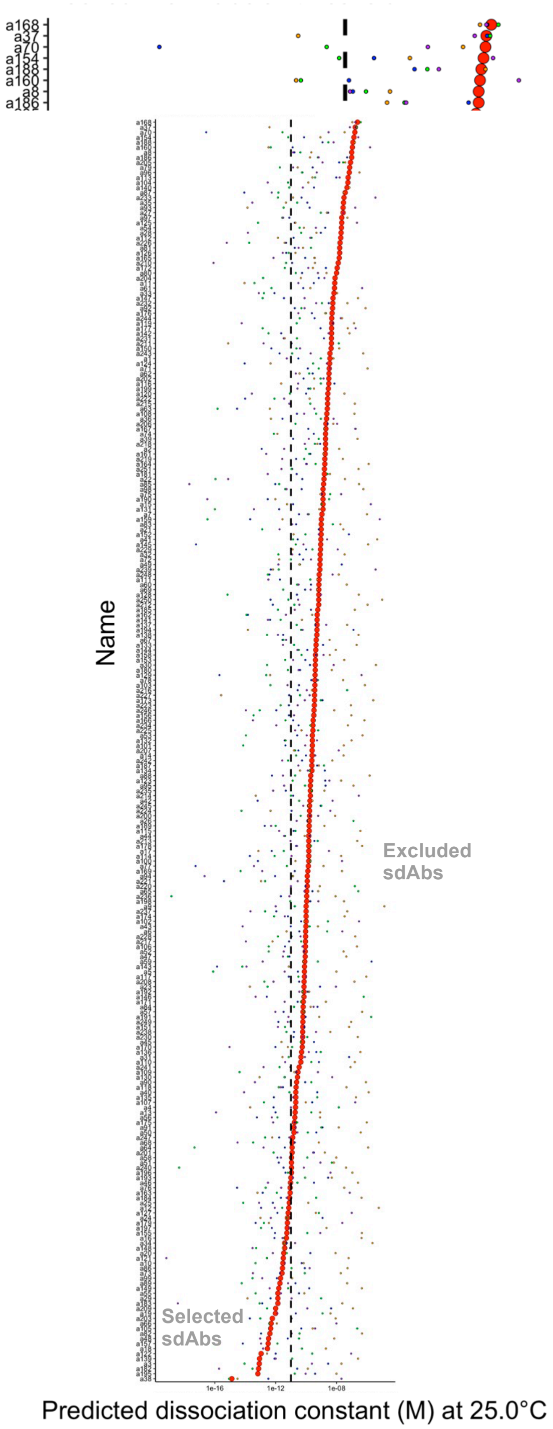

Figure S5. AlphaFold-multimer–Prodigy results. The top 250 sdAbs were modeled with VEEV E2 glycoprotein using AlphaFold-multimer. These predicted complex structures were evaluated with Prodigy for predicted dissociation constants. SdAbs are ordered from high to low dissociation constant for top ranked AlphaFold-multimer models. Dashed line indicates the selected 1 x 10^{-11} M dissociation constant threshold used for down selection, yielding 41 sdAbs (bottom of plot). Ranking determined by AlphaFold

pLDDT metric. Red points are first rank models, with the other colored points indicating the other models rank 2 through 5.

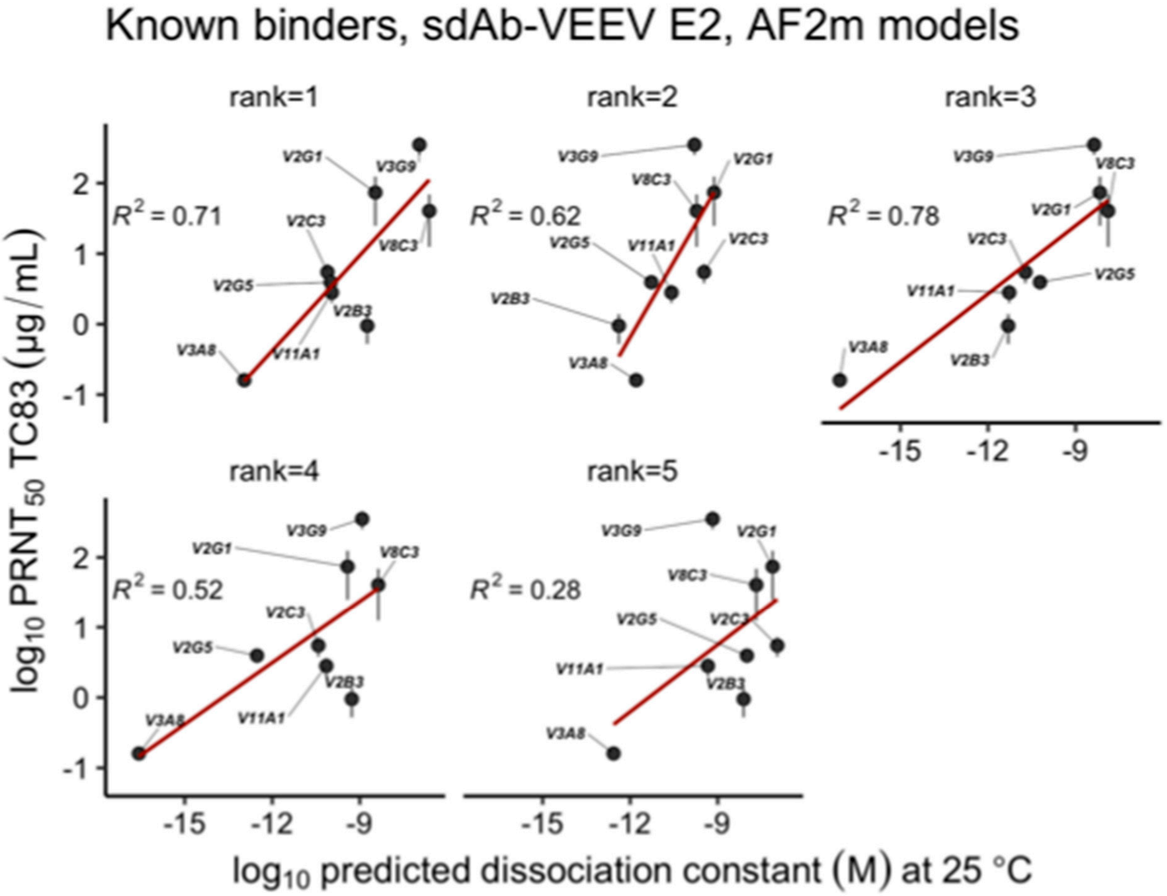

Figure S6. PRNT50 and predicted dissociation constant of sdAbs against VEEV E2 using AF2m models. PRNT50 values of the eight previously identified VEEV-neutralizing sdAbs from Liu *et al.* 2022 [3] plotted against their corresponding Prodigy-predicted dissociation constants when modeled in complex with E2 glycoprotein of VEEV using AlphaFold2-multimer. Each of the five ranked generated models produced are shown for completeness. R-squared values (squared Pearson correlation coefficient) are for best linear fit (dark red line). Error bars indicate standard deviation of experimental PRNT50 values.

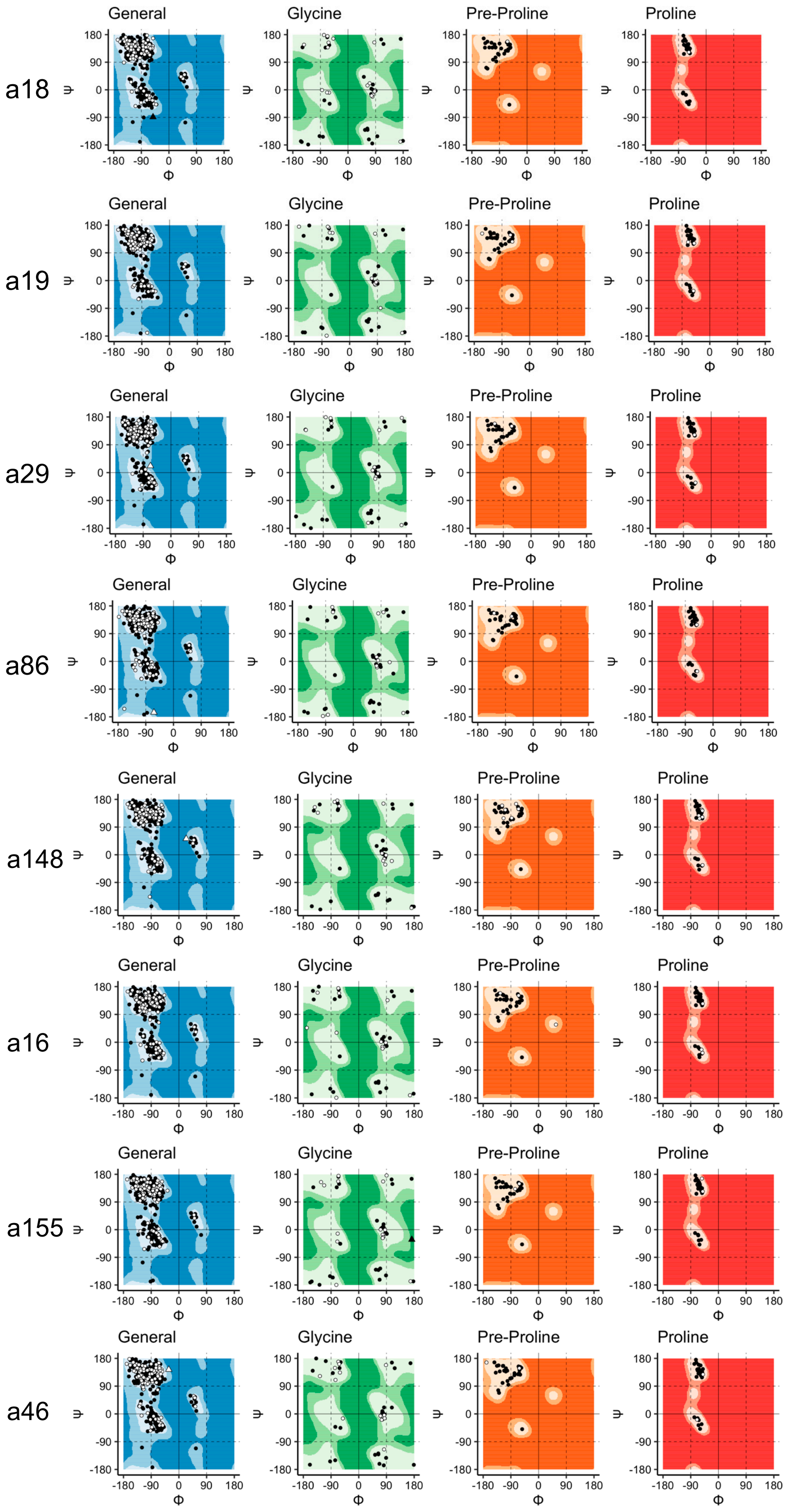
General
Glycine
Pre-Proline
Proline
a18
a19
a29
a86
a148
a16
a155
a46
180
90
0
-90
-180
Ψ
Φ

Figure S7. Ramachandran plots. Ramachandran plots of AlphaFold-multimer sdAb-VEEV E2 complexes were produced for top-ranked structures. Plots are separated by glycine residues (green), pre-proline residues (orange), and proline residues (red), with the remainder of the calculated phi-psi angles placed in general (blue). Normal range of phi-psi angle distributions (lighter coloring) defined by Lovell *et al*. [24]. Color of points are indicative of whether the phi-psi angle is part of the sdAb or VEEV E2 glycoprotein (white for sdAb and black for E2). Shape indicates whether the phi-psi angle falls within the normal range or is an outlier (circle for normal and triangle for outliers).

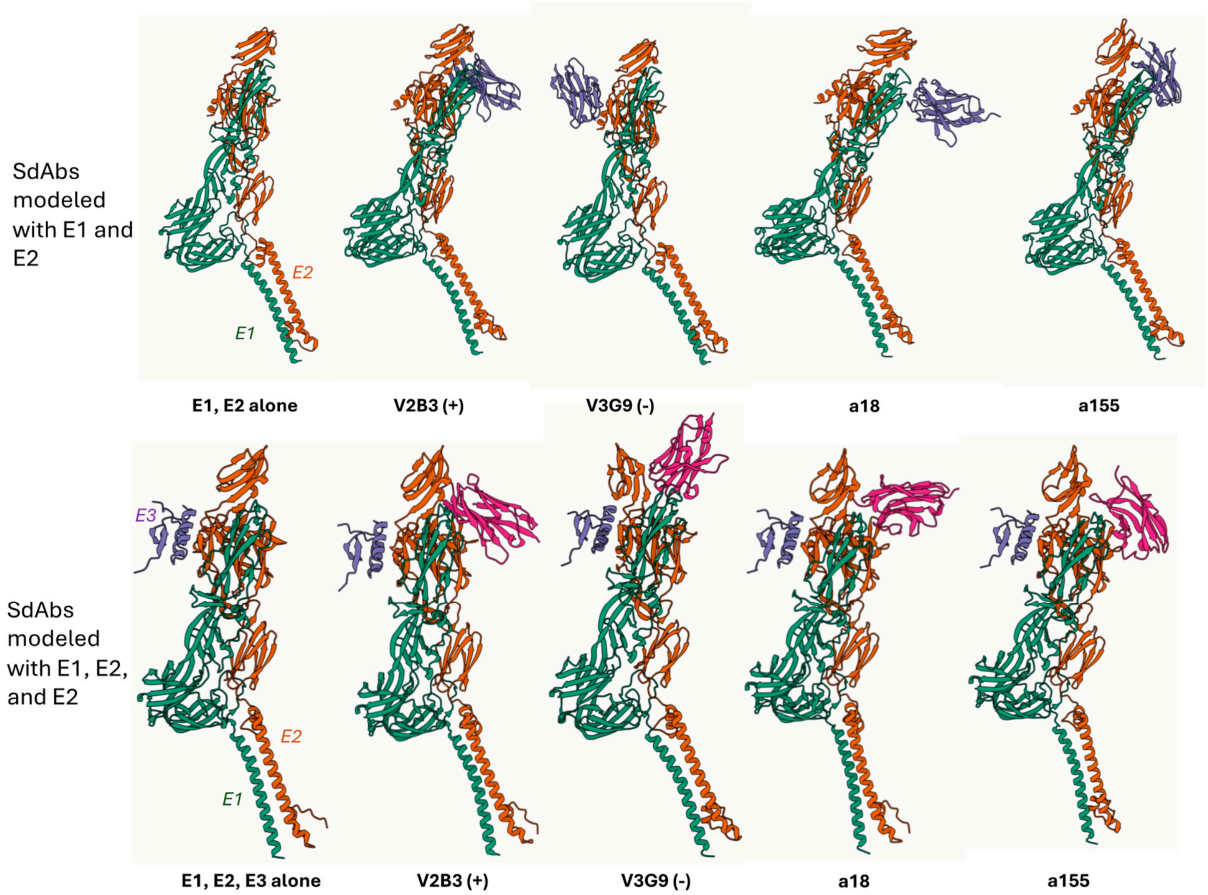

Figure S8. Model comparison between sdAbs. Top) sdAbs were modeled in a complex with E1/E2. E1 is colored green, E2 is colored orange, and sdAbs are colored purple. Bottom) sdAbs were modeled in a complex with E1/E2/E3. E1 is colored green, E2 is colored orange, E3 is colored purple, and sdAbs are colored pink. V2B3 is the positive control, V3G9 is the negative control.

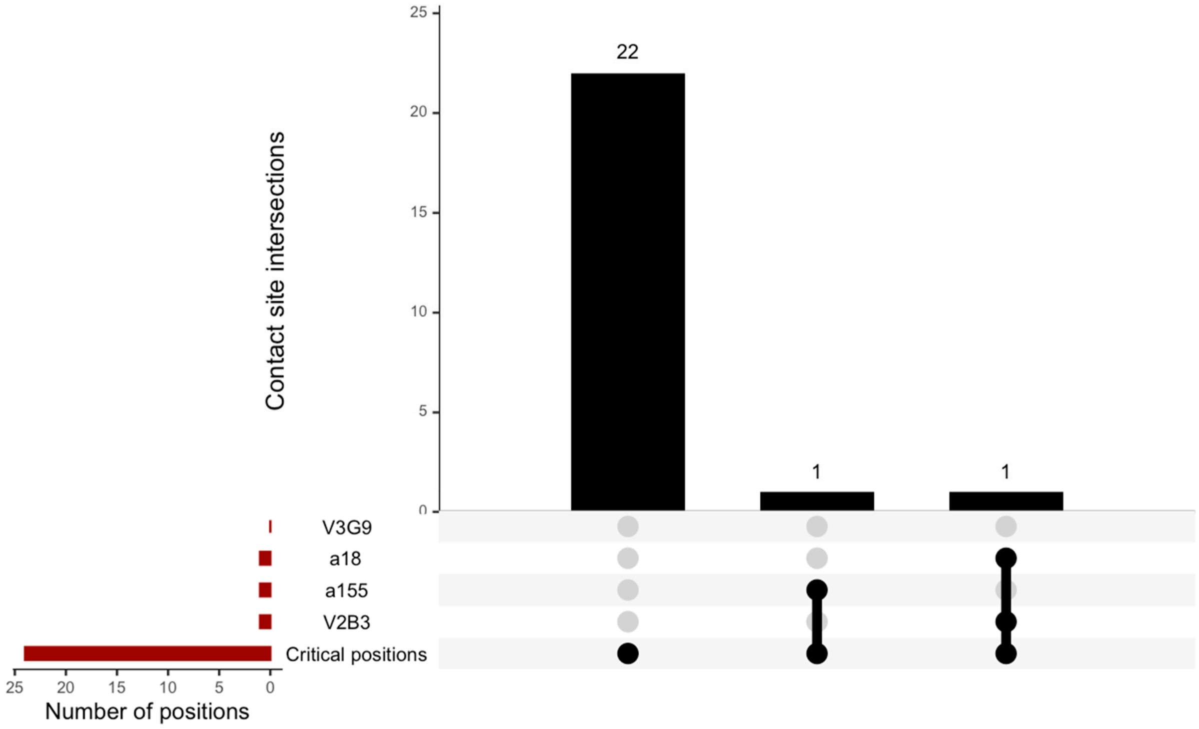

Figure S9. Overlap of residues of E2 critical for neutralizing anti-VEEV mAbs and those in the AlphaFold models. UpSet plot [28] showing the intersections of AlphaFold-predicted contacts of sdAbs with E1/E2/E3 complexes. E2 residues identified as critical for neutralizing by Kafai *et al.* are shown [7].

Combined alphavirus sequence FASTA used for fine-tuning:

>1A12
MAVQLVESGGGLVQAGGSLRLSCAASGRALTSGTMGWFRQAPGKEREFVARANVKGDGTFYAD
AVKGRFTISRDYAKNTVYLQMNNLKPEDTAVYFCAADRGTVYGTSEDLYDYWGQGTQVTVSSG
AR
>1C11
MAVQLVESGGGLVLAGASLRLSCADSGSGRSLGTYGWFRQAPGKEREFVARISQRGGIDYTDSV
KGRFTISRDNSKNTVNLQMTTLTPEDTAVYYCAADPDTVYSRSPALWKYWGQGTQVTVSSGAR
>1D1
MAVQLVESGGGLVQAGGSLRLSCEASGHTFSSGTMGWFRQAPGMEREFVARINEKGDGTFYADS
VKGRFTISRDYAKNTVYLQMNTLKPEDTAVYYCVADRGTVYGTREDLYDYWGQGTQVTVSSGA
R
>1E1
MAVQLVESGGGLVETGGESLRLSCAASGRTIENYHVGWFRQTSGPEREFLAATSRGNGISTIADSVK
GRFTISRDNAKNTVYLQMNALKPEDTAVYICAADDANFVNYRSYRAWDNWGQGTQVTVSSGA
R
>1F1
MAVQLVESGGGLVQAGGSLRLSCAASGRSFSAYTMAWFRQAPGKEREWMASIVRSGGPTYYAD
SVKDRFTISRENARNTVYLQMNSLKPEDTAVYYCAADLGWTYSRSPELFGSWGQGTQVTVSSGA
R
>1G6

MAVQLVESGGGLVQAGGSLRLSCAASGRTFSTYTMGWFRQAPGKEREFVAR11ENTGNIYYADS
VKGRFTISEDNAKNTLYLQMNNLKPEDTAVYYCVGDRGAVYGTRPDLYDHWGQGTQVTVSSGA
R
>1H3
MAVQLVESGGGLVQAGGSLRLSCAASVHTFTMPTMGWFRQAPGKGRELVARFNANINSTYYGD
SVKGRFTISRDFAKNTVYLHMSSLRPEDTAVYYCVADRGVVFGTREDLYDYWGQGTQVTVSSGA
R
>1H7
MAVQLVESGGGLVQAGGSLRISCLASGPPFTDYTMGWFRQAPGKEREFVARITNHGGGTHYADS
VKGRFTISRDYATNTVYLQMNSLKPEDTAVYYCAADRGAVYGTREDLYDYWGQGTQVTVSSGA
R
>2A3
MAVQLVESGGGLVPTGGSLRLSCTASGRTDNLYTVGWFRQAPGKERELIARITEKGGPTYYADSV
KGRFSISTHKSKNAIYLQMNSVKPEDTAVYYCVADRGTVYGTREDLYDYWGQGTQVTVSSGAR
>2B4
MAVQLVESGGGLVQAGGSLRLSCAASGRSSSAYTMAWFRQAPGKEREWMASIVRSGGPTYYAD
SVKDRFTISRENARNTVYLQMNSLKPEDTAVYYCAADLGWTYSRSPELFGSWGQGTQVTVSSGA
R
>2B5
MAVQLVESGGGLVQAGGSLRLSCAASGRTIENYHVGWFRQTSGPEREFLAATSRGNGISTIADSV
KGRFTISRDNAKNTVYLQMNALKPEDTAVYICAADDANFVNYRSYRAWDNWGQGTQVTVSSG
AR
>2E8
MAVQLVESGGGLVQAGGSLRLSCAASGRSFSAYTMAWFRQAPGKEREWMASIVRSGGPTYYAD
SVKDRFTISRENARNTVYLQMNSLKPEDTAVYYCAADLGWTYSRSRELFGSWGQGTQVTVSSG
AR
>2F7
MAVAGESGGGVVQAGGSLRLSCAASGRTFSPYAMGWFRQAPGKERESVAAISWSAGDTYYADS
VKGRFTISRDNAKNTVYLQMNSLKPEDTAVYYCASRNTYFSDSGYSNFYAYDYWGQGTQVTVS
SGAR
>3C5
MAVQLVESGGGLVQAGGSLRLSCAASGRSFSAYTMAWFRQAPGKEREWMASIVRSGGPTYYAD
SVKDRFTISRENARNTAYLQMNSLKPEDTAVYYCAADLGWTYSRSPELFGSWGQGTQVTVSSGA
R
>3E9
MAVQLVESGGGLVQAGGSLSLSCAASGRTFSAGTMGWFRQAPGKERELIARVIKNGGSTYYTDS
VKGRFTIAADFAKSTVYLQMNSLKPEDTALYYCAADRGAVFGTRQELYDDWGQGTQVTVSSGA
R
>4C1
MAVQLVESGGGLVQAGGSLRLSCAASVHTFTMPTMGWFRQAPEKGRELVARFNANINSTYYGD
SVKGRFTISRDFAKNTVYLHMSSLRPEDTAVYYCVADRGVFFGTREDLYDYWGQGTQVTVSSRA
R
>4C7
MAVQLVESGGGLVQAGGSLRLSCATSGRTLGTYGWFRQAPGKEREFVARFLRNNGLTEYADSVK
GRFTISRDDAKNTVELQMIMLKPEDTAVYYCTADPDAVYSRSPALWVYWGQGTQVTVSSGAR
>4F8
MAVQLVESGGGTAQAGGPLRLSCKASGRSFSVGTMAWFRQVPGKEREFLVRDSQKGDGTVYAG
SVEGRFTISRDYAKNTMDLQMNSLKPEDTAVYYCAADRGTVYGTREDLYDYWGQGTQVTVSSG
AR
>4G10

MAVQLVESGGGLVQAGGSLRLSCEASGHTFNSGTMGWFRQAPGMEREFVARINEKGDGTFYAD
SVKGRFTISRDYTKNTVYLQMNTLKPEDTAVYYCVADRGTVYGTREDLYDYWGQGTQVTVSSG
AR
>4H6
MAVQLVESGGGLVQPGGSLGISCVASGPPFTDYTLGWFRQAPGKEREFVARITNHGGGTHYADSV
KGRFTISREYATNTVYLQMNSLKPEDTAVYYCAADRGAVYGTREDLYDYWGQGTQVTVSSGAR
>CC3
EVQLQASGGGSVQAGGSLRLSCVTSQNLFEYYTMGWYRQVPGSQRERVALINNGGSTVAGSVE
GRFTISRDHAKNSVYLQMNYLKPEDSAVYYCRAFGPADYWGQGTQVTVSS
>CG6
EVQLQASGGGLVQPGGSLRLSCVASQNLFEYYTMGWYRQVPGSQRERVALINNGGSNVAGSVEG
RFTISRDNTKNSIYLQMNNLKPEDSAVYYCRAFGPADYWGQGTQVTVSS
>CH5
EVQLQASGGGSVQAGGSLRLSCVASQNLFEYYTMGWYRQVPGSQRERVALINNGDSNVAGSVE
GRFTISRDNAKNSIYLQMNNLKPEDSAVYYCRAFGPADYWGQGTQVTVSS
>CG1
EVQLQASGGGSVQAGGSLRLSCVASQNLFEYYTMGWYRQVPGSQRERVALINNGGSNVAGSVE
GRFTISRDNAKNSIYLQMNNLKPEDSAVYYCRAFGPADYWGQGTQVTVSS
>CC2
EVQLQASGGGSVQAGGSLRLSCVASQNLFEYYTMGWYRQVPGSQRERVALINNGGSNVAGPVE
GRFTISRDNAKNSIYLQMNNLKPEDSAVYYCRAFGPADYWGQGTQVTVST
>CH2
EVQLQASGGGSVQAGGTLRLSCVSSQNLFEYYTMSWYRQVPGSQRERVALINNGGSDVAGSVE
GRFTISRDNAKNSIYLQMNNLKPEDSAVYYCRAFGPADYWGQGTQVTVSS
>CF2
EVQLQASGGGSVQAGGSLRLSCVSSQNLLEYYTMGWYRQVPGSQRERVALINNGGSNVAGSVE
GRFTISRDNAKNSIYLQMNNLKPEDSAVYYCRAFGPADYWGQGTQVTVSS
>CD11
DVQLQASGGGLVQAGGTLRLSCAHSGRTSSTQFWGWFRQAPGKEREFVAGMSRSGLSTFYADS
VKGRFAISRDSGKNTVYLQMNSLKPEDTAVYFCASSPFIGEHYYSSTKYHYWGQGTQVTVSS
>CC12
EVQLQASGGGLVQAGGTLRLSCAHSGRTSSTQFWGWFRQAPGKEREFVAGMSRSGLSTFYADSV
KGRFAISRDNGKNTVYLQMNSLKPEDTAVYFCASSPFIGEHYYSSTKYHYWGQGTQVTVSS
>CB11
DVQLQASGGGLVQAGGTLRLSCAHSGRTSSTQFWGWFRQAPGKEREFVAGMSRSGLSTFYADS
VKGRFAISRDNGKNTVYLQMNSLKPEDTAVYFCASSPFIGEHYYSSTKYHYWGQGTQVTVSS
>CE7
EVQLQASGGGLVQAGGTLRLSCAHSGRTSSTQFWGWFRRAPGKEREFVAGMSRSGLSTFYADSV
KGRFAISRDNGKNTVYLQMNSLKPEDTAVYFCASSPFIGEHYYSSRKYHYWGQGTQVTVSS
>CH6
EVQLQASGGGLVQAGGSLRLSCAASQNIFSINVMGWYRQAPGEQRELVAAITSGGSTNVADSVK
GRVTISRDNAKNTVYLQMNSLKPEDTAVYYCAAEETYYSGSYYGDMEYWGQGTQATVSS
>CA6
EVQLQASGGGLVRPGGSLRLSCAASGSFFTIDTMAWYRQAPGRRRELVARQSSGRSPDYDDSVV
GRFTISRDIAKSSVYLQMDSLQPEDTALYYCYQSIRPWPGSSYEAHWGQGIQVIVSS
>CC5
EVQLQASGGGLVQPGGSLRLSCAASGSFFTIDTMAWYRQAPGKQRELVARQSSGRSPDYDDSVV
GRFTISRDIAKSSVCLQMDSLQPEDTALYYCYQSIRPWPGSSYEAHWGQGIQVIVSS
>V3A8
DVQLQASGGGSVQAGGSLRLSCAASGHSFANYHVAWIRQTPGKECEFVGSASRRDDSTYYADFA
LGRFIISRDNDINTAYLQMNSLKPEDSAVYYCVAAVMAQTTQGWTTDYDLRGQGTQVTVSS

>V2G1
VQLQASGGGLVQAGDSLRLSCAASGRTIKGYAVGWFRQASGKEREFVAVISYFDERADYAHSAE
GRFTISRDNAKDTVVLQMNSLKPEDTAVYFCAAGLSESTLPSEYIYWGQGTQVSVSS
>V1c2
EVQLQATGGGLVQAGGSLKVSCAASGRTFSSLAMAWFREAPGNEREFVAAIMWTGDRTHYADF
VKGRFTISRDNALNTVSLQMNNLKPADTAVYYCAGAFSFPSQFARDYTYWGQGTQVSVSS
>V2b3
EVQLQASGGGLVQAGGSLRLSCAASGSIVSINVMAWYRQSPGKERELVAKSSGPFTLYADSVKGR
FTISNDAAKNTVDLQMNSLKPEDTAVYYCNAEGLRYPSGTYGPSAVWGQGTQVTVSS
>V4f3
EVQLQASGGGAVQIGGSLRLSCTASGRTFSSLAMAWFREAPGNEREFVAAIVWTGDRTHYADFV
KGRFTISRDNALNTVSLQMNNLKPVDTAVYYCAGAFSFPSQFARDYTYWGQGTQVSVSS
>V3e2
EVQLQASGGGLVQAGGSLKVSCAASGRTFSSLAMAWFREAPGNEREFVAAIMWTGDRTHYADF
VKGRFTISRDNALNTVSLQMNNLKPADTAVYYCAGAFSFPSQFARDYTYWGQGTQVSVSS
>V6g1
EVQLQASGGRLVQAGGSLKVSCAASGRTFSSLAMAWFREAPGNEREFVAAIMWTGDRTHYADF
VKGRFTISRDNALNTVSLQMNNLKPADTAVYYCAGAFSFPSQFARDYTYWGQGTQVSVSS
>V7b4
EVQLQASGGGLVGAGGSLRVSCAASGRTFSSLAMAWFREAPGNEREFVAAIMWTGDRTHYADF
VKGRFTISRDNALNTVSLQMNNLKPADTAVYYCAGAFSFPSQFARDYTYWGQGTQVSVSS
>V11a1
DVQLQASGGGSVQAGGSLRLSCVASQNLFEYYTMGWYRQVPGSQRERVALINNGGSNVAGSVE
GRFTISKDNAKNSIYLQMNNLKPEDSAVYYCRAFGPADYWGQGTQVTVSS
>V15c5
DVQLQASGGGSVQAGGSLRLSCVASQNLFEYYTMGWYRQVPGSLRERVALINNGGSNVAGSVE
GRFTISRDNAKNSIYLQMNNLKPEDSAVYYCRAFGPADYWGQGTQVTVSS
>V21a4
EVQLQASGGGLVQAGGSLRLSCAASGSIVSINVMAWYRQSPGKQRELVAKSSGPFTLYADSVKGR
FTISNDAAKNTVDLQMNSLKPEDTAVYYCNAEGLRYPSGTYGPSAVWGQGTQVTVSS
>V22h3
VQLQASGGGLVQAGGSLRLSCAASGSIVSINVMAWYRQSPGKERELVAKSSGPFTLYADSVKGRF
TISNDAAKNTVDLQMNSLKPEDTAVYYCNAEGLRYPSGTYGPSAVWGQGTQVTVSS
>V23e1
VQLQASGGGLVQAGGSLRLSCAASGSIVSINVMAWYRQSPGKQRELVAKSSGPFTLYADSVKGRF
TISNDAAKNTVDLQMNSLKPEDTAVYYCNAEGLRYPSGTYGPSAVWGQGTQVTVSS
>V28a1
VQLQASGGGSVQAGGSLRLSCVASQNLFEYYTMGWYRQVPESQRERVALINNGGSNVAGSVEG
RFTISRDNAKNSIYLQMNNLKPEDSAVYYCRAFGPADYWGQGTQVTVSS
>V31c6
EVQLQASGGGLVQAGGSLRLSCAASGSIVSINVMAWYRQSPGKERELVAKSSGPFTLYADSVKGR
FTISNDAAKNTVDLQMNSLKPEDTAVYYCNAEGLRYPSGTYGPSAVWGQGTQVTVSS
>V32a5
VQLQASGGGLVQAGGSLKVSCAASGRTFSSLAMAWFREAPGNEREFVAAIMWTGDRTHYADFV
KGRFTISRDNALNTVSLQMNNLKPADTAVYYCAGAFSFPSQFARDYTYWGQGTQVSVSS
>V33g6
DVQLQASGGGSVQAGGSLRLSCVASQNLFEYYTMGWYRQVPESQRERVALINNGGSNVAGSVE
GRFTISRDNAKNSIYLQMNNLKPEDSAVYYCRAFGPADYWGQGTQVTVSS
>V2C4
DVQLQASGGGSVQAGGSLRLSCVASQNLFEYYTMGWYRQVPGSQRERVALINNGGSNVAGSVE
GRFTISRDNAKNSIYLQMNNLKPEDSAVYYCRAFGPADYWGQGTRVSVSP

>V2E4
VQLQASGGRLVQAGGSLKVSCAASGRTFSSLAMAWFREAPGNEREFVAAIMWTGDRTHYADFV
KGRFTISRDNALNTVSLQMNNLKPADTAVYYCAGAFSFPSQFARDYTYWGQGTQVSVSS
>V2H5
DVQLQASGGGTVQAGASLRPSCAATGRTFSSLAMAWFREAPGNEREFVAAIMWTGDRTHYADF
VKGRFTISRDNALNTVSLQMNNLKPADTAVYYCAGAFSFPSQFARDYTYWGQGTQVSVSS
>V2H6
DVQLQASGGGSVQAGGSLRLSCVASQNLFEYYTMGWYRQVPESQRERVALINNGGSNVAGSVE
GRFTISRDNAKNSIYLQMNNLKPEDSAVYYCRAFGPADYWGQGTQVTVSS
>V3B7P2
EVQLQASGGGLVQAGGSLKVSCAASGRTFSSLAMAWFREAPGNEREFVAAIMWTGDRTHYADF
VKGRYTISRDNALNTVSLQMNNLKPADTAVYYCAGAFSFPSQFARDYTYWGQGTQVSVSS
>V3B11
DVQLQASGGGLVQAGGSLRLSCAASGSIVSINVMAWYRQSPGKERELVAKSSGPFTLYADSVKGR
FTISNDAAKNTVDLQMNSLKPEDTAVYYCNAEGLRYPSGTYGPSAVWGQGTQVTVSP
>V3E7P2
EVQLQASGGGLVQAGGSLKVSCAASGRTFSSLAMAWFREAPGNEREFVAAIMWTGDRTHYADF
VKGRFTISRDNALNTVSLQMNNLKPTDTAVYYCAGAFSFPSQFARDYTYWGQGTQVSVSS
>V3E10P2
EVQLQASGGGLVQAGGSLKVSCAASGRTFSSLAMAWFREAPGNEREFVAAIMWTGDGTHYADF
VKGRFTISRDNALNTVSLQMNNLKPADTAVYYCAGAFSFPSQFARDYTYWGQGTQVSVSS
>V3G9P2
EVQLQASGGGLVQAGGSLKVSCAASGRTFNNLAMAWFREAPENEREFVAAIMWTGDRTHYADF
VKGRYTISRDNALNTVSLQMNNLKPADTAVYYCAGAFSFPSQFARDYTYWGQGTQVSVSS
>V3G10P2
EVQLQASGGGLVQAGGSLKVSCAASGRTFSSLAMAWFREAPGDEREFVAAIMWTGDRTHYADF
VKGRFTISRDNALNTVSLQMNNLKPADTAVYYCAGAFSFPSQFARDYTYWGQGTQVSVSS
>V2C3P1
DVQLQASGGGSVQAGGSLRLSCVASQNLFEYYTMGWYRQVPGSQRERVALINNGGSNVAGSVE
GRFTISRDNAKNSIYLQMNNLKPEDSAVYYCRAFGPADYWGQGTQVTVSS

BLASTp (protein-protein BLAST) results for each of the sdAbs:

```
sdAb: a18
Top hit: surface immunoglobulin M heavy chain variable region, partial [Vicugna
pacos] Sequence ID: CAQ53184.1
Score   Expect  Method  Identities  Positives   Gaps
199  bits(506)     4e-61     Compositional  matrix  adjust.       98/123(80%)
107/123(86%)    2/123(1%)

Query  3    VQLVESGGGLVQAGGSLRLSCAASGFIFGRNAMGWYRQAPGKEREGVSCISSGGGRTSYA  62
            +QLVESGGGLVQAGGSLRLSCAASGF F   A+GW+RQAPGKEREGVSCISS  G T YA
Sbjct  2    LQLVESGGGLVQAGGSLRLSCAASGFTFDDYAIGWFRQAPGKEREGVSCISSSDGSTYYA  61

Query  63   DSVKGRFTISRDNAKNTVYLQMNNLRAEDTAIYYCAA--AGYGQELSYHYDYWGQGTQVT  120
            DSVKGRFTIS DNAKNTV+LQMN+L+ EDTA+YYCAA  AG G   SY++DYWGQGTQVT
Sbjct  62   DSVKGRFTISSDNAKNTVHLQMNSLKPEDTAVYYCAADLAGLGCSGSYYHDYWGQGTQVT  121

Query  121  VSS  123
            VSS
Sbjct  122  VSS  124
```

sdAb: a19
Top hit: immunoglobulin heavy chain variable region, partial [Homo sapiens]
Sequence ID: QRN76808.1
Score Expect Method Identities Positives Gaps
182 bits(462) 1e-56 Compositional matrix adjust. 90/116(78%)
98/116(84%) 2/116(1%)

```
Query  4    VQVVESGGGLMQAGGSLRLSCAASGFPVDSAHMHWVRQAPGKGLEWIGEIHHSGSTYYNP  63
            VQ+VESGGGL+Q GGSLRLSCAASGF V S +M WVRQAPGKGLEW+  I+  GSTYY
Sbjct  2    VQLVESGGGLVQPGGSLRLSCAASGFTVSSNYMSWVRQAPGKGLEWVSLIYSGGSTYYAD  61

Query  64   SLKSRVTISRDNSKNTLYLQMNNLRAEDTAIYYCAVDTF--GADYWGQGTQVTVSS  117
            S+K R TISRDNSKNTLYLQMN+LRAEDTA+YYCA DTF  G DYWGQGT VTVSS
Sbjct  62   SVKGRFTISRDNSKNTLYLQMNSLRAEDTAVYYCARDTFGRGGDYWGQGTLVTVSS  117
```

sdAb: a29
Top hit: immunoglobulin heavy chain VHDJ region, partial [Camelus dromedarius]
Sequence ID: BAD00520.1
Score Expect Method Identities Positives Gaps
186 bits(472) 5e-58 Compositional matrix adjust. 92/119(77%)
103/119(86%) 1/119(0%)

```
Query  3    VQLVESGGGLVQAGGSLRLSCAASGYDFSSYWMHWVRQAPGKALEWVSGISDHGY-TNYA  61
            VQLVESGGGLVQ GGSLRLSCAASG+ FS +WMHWVRQAPGK LEWVSGI+  G  T YA
Sbjct  2    VQLVESGGGLVQPGGSLRLSCAASGFTFSKFWMHWVRQAPGKGLEWVSGINPVGINTYYA  61

Query  62   DSVKGRFTISRDNAKNTVYLQMNSLQPEDTGVYYCAARYGNTLAYYDYWGQGTQVTVSS  120
            DSVKGRFTISRDNAKNT+YLQ++SL+ EDT +YYCA  + +  +YYDYWGQGTQVTVSS
Sbjct  62   DSVKGRFTISRDNAKNTLYLQLDSLKIEDTAMYYCANLFPSGDSYYDYWGQGTQVTVSS  120
```

sdAb: a86
Top hit: Chain B, Nanobody [Camelidae] Sequence ID: 5IMK_B
Score Expect Method Identities Positives Gaps
190 bits(482) 2e-59 Compositional matrix adjust. 95/124(77%)
103/124(83%) 6/124(4%)

```
Query  3    IQLVESGGGLVQAGGSLRLSCAASGRTFSSLAMAWFREAPGKEREWVAAIVWTGDRTHYA  62
            +QLVESGGGLVQAGGSLRLSCAASGRTFSS  M WFR+APGKERE+VAAI W G  T+YA
Sbjct  2    VQLVESGGGLVQAGGSLRLSCAASGRTFSSYGMGWFRQAPGKEREFVAAIRWNGGSTYYA  61

Query  63   DFVKGRFTISRDNALNTVSLQMNNLKPVDTAVYYCAAGR------SLLSDYAYWGQGTQV  116
            D VKGRFTISRDNA NTV LQMN+LKP DTAVYYCAAGR      S   +Y YWGQGTQV
Sbjct  62   DSVKGRFTISRDNAKNTVYLQMNSLKPEDTAVYYCAAGRWDKYGSSFQDEYDYWGQGTQV  121

Query  117  SVAS  120
            +V+S
Sbjct  122  TVSS  125
```

sdAb: a148
Top hit: Chain D, Nanobody 8A2 [Camelus dromedarius] Sequence ID: 7TPR_D
Score Expect Method Identities Positives Gaps

```
185  bits(469)      2e-57     Compositional  matrix  adjust.       96/128(75%)
103/128(80%)    5/128(3%)

Query  2    VQLQASGGGSVQAGGSLRLSCTASESTFDLYVMGWFRQAPGKGPEWVSGINADGSNTHYT  61
            VQL  SGGGSVQAGGSLRLSC AS  T+ +  MGW+RQAPG+G EWVSGINADGSNTHYT
Sbjct  2    VQLVDSGGGSVQAGGSLRLSCAASGYTYSICTMGWYRQAPGEGLEWVSGINADGSNTHYT  61

Query  62   DSVKGRFTISRDNSKNTLYLQMNALKPEDTAVYYCAA----DPATPGSEKPAYHYDYWGQ  117
            DSVKGRFTISRDN+K TLYLQMN+LKPEDTA+YYCAA    D   P     A  Y YWGQ
Sbjct  62   DSVKGRFTISRDNAKKTLYLQMNSLKPEDTAIYYCAAHGTYDKYAP-CGGFAGTYTYWGQ  120

Query  118  GTQVTVSS  125
            GTQVTVSS
Sbjct  121  GTQVTVSS  128
```

sdAb: a16
Top hit: Chain B, Camelid-Derived Antibody Fragment Nb22 [Vicugna pacos] Sequence ID: 5LHR_B

```
Score   Expect  Method  Identities  Positives   Gaps
192  bits(487)      4e-60     Compositional  matrix  adjust.       98/122(80%)
105/122(86%)    3/122(2%)

Query  1    EVQLVESGGGLVQAGGSLRLSCVASRRLFEYYTMGWYRQAPGKQREFVAAISWSGGGSSY  60
            +VQL ESGGGLVQAGGSLRLSC AS R F  Y MGW+RQAPGK+REFVAAISWSGG ++Y
Sbjct  1    QVQLQESGGGLVQAGGSLRLSCAASGRTFSSYVMGWFRQAPGKEREFVAAISWSGGSTNY  60

Query  61   ADSVKGRFTISRDNAKNTVYLQMNNLKPEDSAVYYCAADPALRYSDS---WGQGTQVTVS  117
            ADSVKGRFTISRDNAKNTVYLQMN+LKPED+AVYYCAAD A     S   WGQGTQVTVS
Sbjct  61   ADSVKGRFTISRDNAKNTVYLQMNSLKPEDTAVYYCAADLASSRDVSSWYWGQGTQVTVS  120

Query  118  SA  119
            SA
Sbjct  121  SA  122
```

sdAb: a155
Top hit: surface immunoglobulin M heavy chain variable region, partial [Vicugna pacos] Sequence ID: CAQ53179.1

```
Score   Expect  Method  Identities  Positives   Gaps
205  bits(521)      2e-63     Compositional  matrix  adjust.      103/124(83%)
107/124(86%)    8/124(6%)

Query  3    VQLVESGGGLVQAGGSLRLSCAASGRTFSAGTMGWFRQAPGKEREFVAAIRWSGGSAYYA  62
            VQLVESGGGLVQAGGSLRLSCAASGRTFS+  MGWFRQAPGKEREFVAAI WSGGS YYA
Sbjct  2    VQLVESGGGLVQAGGSLRLSCAASGRTFSSYAMGWFRQAPGKEREFVAAISWSGGSTYYA  61

Query  63   DSVKGRFTISRDNGKNTVYLQMNSLKLEDTAVYYCASSRPGTV--------NYWGKGTQV  114
            DSVKGRFTISRDN KNTVYLQMNSLK EDTAVYYCA+ R  TV        +YWG+GTQV
Sbjct  62   DSVKGRFTISRDNAKNTVYLQMNSLKPEDTAVYYCAADRSYTVVAGPRYEYDYWGQGTQV  121

Query  115  TVSS  118
            TVSS
Sbjct  122  TVSS  125
```

sdAb: a46

```
Top hit: Chain E, M170 Nab [Lama glama] Sequence ID: 7DST_E
Score   Expect  Method  Identities  Positives   Gaps
172  bits(435)      2e-52      Compositional  matrix  adjust.       86/118(73%)
97/118(82%)     1/118(0%)

Query  1    EVQLQASGGRLVQAGGSLRLSCAASGRTFSAGTMGWFRQAPGKQREAVSAIAGTDG-IYY  59
            +VQLQ SGG LVQAGGSLRLSCAASGRTFS+  MGWFRQAPG +RE V+ I+ + G  YY
Sbjct  4    QVQLQESGGGLVQAGGSLRLSCAASGRTFSSYAMGWFRQAPGSEREFVARISWSGGSTYY  63

Query  60   TDSVKGRFTISRYNAKNTAYLQMNNLRAEDTAIYYCNIGRVTPYADFWGQGTQVTVSS  117
             DSVKGRFTISR NAKNT YLQMN+L+ EDTA+YYC  G   P +D+WGQGTQVTVSS
Sbjct  64   ADSVKGRFTISRDNAKNTVYLQMNSLKPEDTAVYYCTAGFALPPSDYWGQGTQVTVSS  121
```

CDR3s of the sdAbs using IMGT numbering:

```
a18  AGYGQE---LSYHYDY
a19  DTF---------GADY
a29  RYGNT-----LAYYDY
a86  GRSL------LSDYAY
a148 DPATPGSEKPAYHYDY
a16  DPAL-------RYSDS
a155 SRP--------GTVNY
a46  GRVT-------PYADF
```
